# The Impact of Predialysis Patient Education Counseling on Relative Frequencies of Renal Replacement Modalities

**DOI:** 10.7759/cureus.10834

**Published:** 2020-10-07

**Authors:** Naveed Mirza, Khalid A Sheikh, Saad Muzaffar, Noureen Chaudary, Dildar Ahmed, Ishtiaque Alam

**Affiliations:** 1 Nephrology, Armed Forces Hospital Southern Region, Khamis Mushait, Khamis Mushait, SAU; 2 Neonatology, Armed Forces Hospital Southern Region, Khamis Mushait, Khamis Mushait, SAU; 3 Nephrology, Rawalpindi Medical University, Rawalpindi, PAK

**Keywords:** chronic kidney disease, end-stage renal disease, renal replacement therapy, pre-dialysis education program, hemodialysis

## Abstract

Background and objective

The predialysis education program (PDEP) is an integral part of the management of patients with end-stage renal disease (ESRD). Hence, the aim of this study was to assess the distribution of renal replacement therapy (RRT) among patients with ESRD who received PDEP counseling at a single tertiary care center in Khamis Mushait, Kingdom of Saudi Arabia (KSA).

Methodology

In this study, we included 177 patients with ESRD who received a series of structured PDEP counseling sessions between March 2018 and February 2019 at the Armed Forces Hospital, Southern Region, Khamis Mushait, KSA. All patients were offered available RRT options, which included hemodialysis (HD), continuous ambulatory peritoneal dialysis (CAPD), or renal transplantation. Patients' opted RRT modality was recorded and managed as per the international guidelines and institutional protocols.

Results

Out of 177 patients, 58.8% (104) were males, with a mean age of 59 ± 13.7 years. The most common comorbid condition as the primary diagnosis was diabetic nephropathy in 49.7% (88), followed by hypertension in 30.5% (54), bilateral small kidney in 15.3% (27), and renal stone in 5.1% (nine) of the patients. Among the available RRTs, 51.4% (91) chose HD, 5.1% (nine) decided on CAPD, four patients (2.3%) opted for renal transplantation, while the remaining 41.2% (73) had yet to choose one, out of which 83.6% (61/73) had stage-IV chronic kidney disease (CKD) while the remaining 16.4% (12/73) had stage-V CKD.

Conclusions

In conclusion, a series of structured PDEP sessions for the patients progressing to ESRD can facilitate their selection of RRT. In a resource-limited setting, such as ours, where the number of patients who seek treatment for ESRD is rising, PDEP can provide patients with adequate information and knowledge to equip them with the ability regarding the selection of a self-care RRT modality.

## Introduction

Kidney failure is a global public health problem, affecting more than 750 million individuals worldwide [1]. It contributes significantly to the global annual mortality and morbidity rates as a non-communicable disease (NCD) [2]. According to a conservative estimate, at this point in time, more than 2.5 million patients are on renal replacement therapies (RRT), and by the year 2030, the number patients receiving treatment for end-stage kidney disease is expected to increase by a factor of more than two, to an estimated number of 5.4 million patients [2,3]. RRT is the one available lifesaving treatment option for patients with end-stage kidney disease, either in the form of dialysis or renal transplantation [3]. Due to its substantial cost, the availability of RRT varies widely between developed and developing countries. It has been available for more than 50 years in developed countries; however, making RRT available to all patients remains a challenge in low- and middle-income countries due to economic constraints as well as the high prevalence of end-stage kidney disease in these countries [1,3,4]. Disparities between developed and developing countries are not only limited to the availability of RRT, but are also observed across the spectrum of disease management, from creating awareness to prevention, screening, care, and treatment [5,6].

An increasing trend in the number of patients with end-stage renal disease (ESRD) has been witnessed worldwide in the past few years, and it is expected to continue for the foreseeable future. This trend can be partly explained by the increase in the aging population and the increasing prevalence of risk factors such as type II diabetes mellitus [7-11]. Patient education and early referral are the two clinically endorsed methods of slowing the progression, and the prevention and detection of ESRD [12].

Considering the complexities involved in the progression and management of the disease, a shared decision-making process that involves empowering the patients and their families is necessary for the management of chronic kidney disease (CKD) patients [9]. Patient education interventions have been reported to be effective not only in enhancing disease-related knowledge but also in curtailing the overall progression of the disease, selection of renal replacement modality (RRM), and enhancing overall outcome and survival rates and the quality of life [7-9,12,13]. Hence, the predialysis education program (PDEP) is an integral part of the management and treatment of patients with ESRD. The intent of PDEP is to empower the patients for shared decision-making by equipping them with the required knowledge on the disease process as well as skills and motivation to deal with the treatment, along with providing information regarding potential treatment options, diet, and drug prescriptions [8,9,14].

The distribution of the choice of RRT among patients with CKD significantly varies depending on their country of origin. These differences have been shaped by various factors such as available treatment options, financial situations, social mores, access to the dialysis centers, and availability of skills required for the particular treatment modality [14]. Kingdom of Saudi Arabia (KSA) is a land of cultural and economic diversities, and hence patients presenting to our resource-limited center belong to a wide range of socioeconomic classes. All CKD patients progressing to ESRD receive a series of structured PDEP counseling sessions. The aim of this study was to assess the distribution of RRT among patients with ESRD who received PDEP sessions at a single tertiary care center in Khamis Mushait, KSA.

## Materials and methods

This study was initiated after obtaining approval from the institutional ethical review committee. In this study, we included 177 patients who presented with CKD stage IV to ESRD between March 2018 and February 2019 at the Armed Forces Hospital, Southern Region, Khamis Mushait, KSA. After obtaining informed consent, adult patients of either gender between the ages of 18-90 years were included in the study. Patients on dialysis after renal graft failure and patients who transferred to our center after the initiation of RRT from any other center were not included in this study. Data regarding demographic and baseline characteristics such as gender, age, comorbid conditions, diagnosis, and serology were obtained.

All patients were offered the available RRT options at our center, which included hemodialysis (HD), continuous ambulatory peritoneal dialysis (CAPD), or renal transplantation (either cadaveric or from a related living donor). All patient were assessed by experienced nephrologists; after clinical evaluation and the assessment of comorbidities and creatinine clearance (CrCl), patients as well as family/guardians of the patients progressing to ESRD with CKD stage IV as per the Kidney Disease Improving Global Outcomes (KDIGO) clinical practice guideline [15] received a series of structured PDEP counseling sessions.

The PDEP comprises a detailed session on self-care treatment modalities, followed by an informative video session on each of the available RRT modalities (CAPD and HD). A brochure was provided to the patients and their families after the video session, covering all the main points of the video session. All patients were offered a chance to speak with other patients receiving treatment to get first-hand knowledge about the experience of certain treatment modalities. All patients were also offered a tour of the in-center unit. Patients who opted for RRT were recorded and managed as per the guidelines and institutional protocols.

Collected data were analyzed with IBM SPSS Statistics version 19 (IBM, Armonk, NY). Results were expressed as mean ± standard deviation (SD) and frequency counts (%) for quantitative and qualitative variables respectively. The association between patients’ choice of RRT and demographic and clinical characteristics were assessed by applying the Chi-square test, and a p-value of ≤0.05 was considered statistically significant.

## Results

During the study period of 12 months (March 2018 to February 2019), a total of 177 patients progressing to ESRD received a series of structured PDEP counseling sessions. Of these patients, 58.8% (104) were male, with a mean age of 59 ± 13.7 years, ranging from 19 to 90 years of age. Eight (4.5%) patients were positive for hepatitis B/C, and the most common primary diagnosis was diabetic nephropathy, which was observed in 49.7% (88) of the patients, followed by hypertension in 30.5% (54), bilateral small kidney in 15.3% (27), renal stone in 5.1% (nine), and various other ailments in 5.1% (nine) patients. Of these nine patients, three had vesicoureteral reflux, two had lupus nephritis, two had immunoglobulin A (IgA) nephropathy, one had focal segmental glomerulosclerosis (FSG), and one had Nephrops syndrome. Ten (5.6%) of these patients had hypertension as a primary diagnosis along with bilateral small kidneys. Demographic characteristics and primary diagnosis by patients’ choice of RRT are presented in Table 1.

**Table 1:** Demographic characteristics and primary diagnosis stratified by patients’ choice of renal replacement therapy SD: standard deviation; CAPD: continuous ambulatory peritoneal dialysis; FSG: focal segmental glomerulosclerosis; IgA: immunoglobulin A

Characteristics	Renal replacement therapy				P-value
	Hemodialysis	CAPD	Renal transplant	Not decided	
Total, n	91	9	4	73	-
Gender, % (n)					
Male	56% (51)	55.6% (5)	50% (2)	63% (46)	0.804
Female	44% (40)	44.4% (4)	50% (2)	37% (27)	
Age in years					
Mean ± SD	59.53 ± 14.48	59.11 ± 14.73	59.49 ± 11.88	36.5 ± 5.2	-
≤40 years, % (n)	9.9% (9)	11.1% (1)	75% (3)	6.8% (5)	0.002
41-60 years	41.8% (38)	44.4% (4)	25% (1)	49.3% (36)	
>60 years, % (n)	48.4% (44)	44.4% (4)	0% (0)	43.8% (32)	
Hepatitis B/C, % (n)	3.3% (3)	11.1% (1)	0% (0)	5.5% (4)	0.667
Primary diagnosis, % (n)					
Hypertension	29.7% (27)	11.1% (1)	25% (1)	34.2% (25)	0.539
Diabetic nephropathy	53.8% (49)	66.7% (6)	50% (2)	42.5% (31)	0.363
Bilateral small kidney	6.6% (6)	22.2% (2)	25% (1)	24.7% (18)	0.012
Renal stone	5.5% (5)	0% (0)	0% (0)	5.5% (4)	0.861
Other diagnoses, % (n)					
FSG	1.1% (1)	0% (0)	0% (0)	0% (0)	-
IgA nephropathy	2.2% (2)	0% (0)	0% (0)	0% (0)	
Lupus nephritis	1.1% (1)	0% (0)	0% (0)	1.4% (1)	
Nephrops syndrome	0% (0)	0% (0)	0% (0)	1.4% (1)	
Vesicoureteral reflux	1.1% (1)	0% (0)	0% (0)	2.7% (2)	

**Figure 1:**
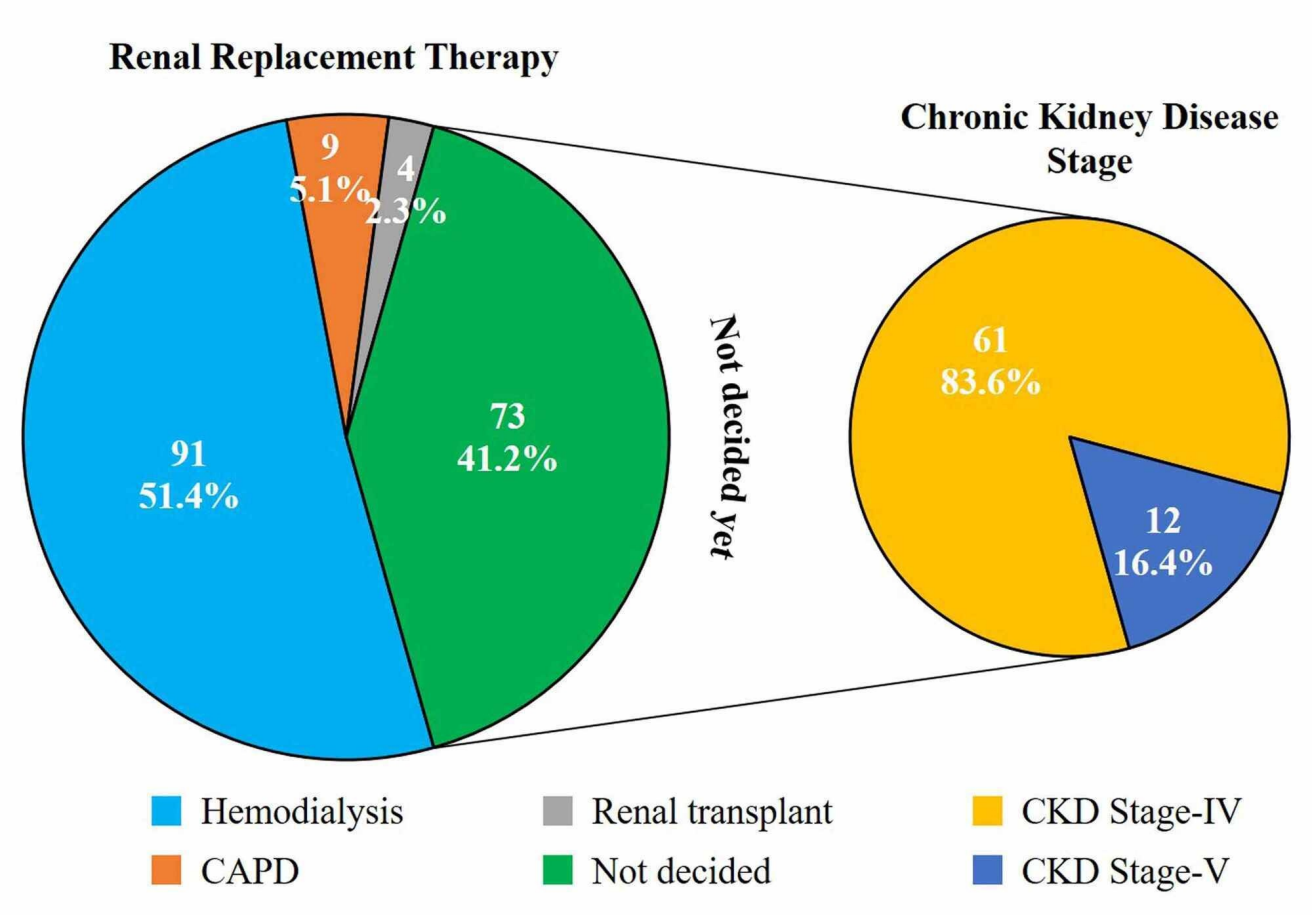
Distribution of patients’ choice of renal replacement therapy CAPD: continuous ambulatory peritoneal dialysis; CKD: chronic kidney disease

Among the available RRTs, 51.4% (91) patients decided on HD, 5.1% (nine) chose CAPD, four (2.3%) opted renal transplantation, while the remaining 41.2% (73) had yet to make a decision, out of which 83.6% (61/73) had CKD stage-IV, while the remaining 16.4% (12/73) had CKD stage-V. The distribution of the patients’ choice of RRT is presented in Figure 1.

## Discussion

PDEP is an integral part of the management and treatment of patients progressing to ESRD; hence, in this study, we assessed the choice of RRT among 177 patients who went through a series of structured PDEP sessions at our center during our observation period of one year. A majority (51.4%) of the patients opted for HD while 41.2% had yet to reach a decision on their choice of RRT. In the past few years, it has been acknowledged that the involvement of patients and their families in healthcare decision-making, as long as it is meaningful, has a profound impact on disease management. It has also been shown to enhance the sense of self-efficacy and autonomy among the patients [16,17]. For patients, various factors play a role in shaping their healthcare decisions; while some of these factors are non-modifiable, some are modifiable, such as education and provision of adequate knowledge [13]. However, a study conducted in the UK reported that most of the written material, such as brochures and flyers, provided to patients at renal centers are hard to follow, especially for those patients with limited educational background [18]. Hence, in recent years, various improvements have been made in this field, which includes the implementation of PDEP for patients progressing to ESRD. However, educating patients alone is not enough, but the timely provision of required knowledge is also important so that they can weigh their treatment options and make an informed decision [13]. Hence, it has been recommended that the educational programs for patients and their families should commence when the glomerular filtration rate (GFR) drops by 30 mL/min [19], as opposed to the previously suggested 20-25 ml/min [14].

Similar to our study, various other studies have also shown that structured PDEP sessions can influence the selection of self-care treatment modalities. In a study conducted by Chanouzas et al. [13], 70% of the patients who attended PDEP sessions chose HD instead of peritoneal dialysis (PD) or conservative management. Another study by Goovaerts et al. [14] has reported that 66% of the patients opted for either in-center or self-care satellite or home HD and 30% chose PD while 4% opted pre-emptive transplantation. It has been observed that the selection of PD as a treatment modality is predominantly driven by the notion of treatment fitting in well with the lifestyle of the patient [20]. Nephrologists believe that self-care modalities are underused worldwide; accordingly, a report by British nephrologists’ has suggested an ideal scenario where 27% of patients should be dialyzed using hospital-based HD, 24% in a satellite unit, 11% using home HD, and 38% on some form of PD [21]. Among other factors, young patients, patients with fewer comorbid conditions, those who are married or living with a partner, and those who are educated or employed are more likely to choose PD over other modalities [13]. However, the CAPD group of patients in our study was comparatively very small, and therefore, no decisive conclusion can be drawn regarding the association between the choice of CAPD and underlying demographic and clinical characteristics. Patients' perception is an integral factor for the choice of modality; for example, it was observed in a study that the patient perception of the ability to cope was more closely related to the choice of PD, and patients were more likely to choose self-care modalities if they found freedom and lifestyle advantages in those modalities [13].

The multi-dimensional educational programs for predialysis patients have also been found to have positive effects on the progression of the disease. Programs that provide not only basic knowledge but also coping skills, emotional support, motivation, and encouragement can lead to delay in the progression of the disease and even possible prevention [8]. The multidisciplinary predialysis education (MPE) is also reported to be associated with a significant reduction in post-dialysis inpatient and total medical expenditures [7]. Noncompliance with CKD educational modules is one of the challenges faced in establishing the efficacy of education programs. A study has reported that only 28% completed the modules, and financial constraints among patients, the severity of illness, lack of understanding, and low healthcare priority were the main drivers of poor compliance [12]. There was no conflict of interest.

To the best of our knowledge, this the first study from KSA regarding the implementation of PDEP and its impact on patients' choice of RRT. An important limitation of this study is that a significant number of patients had yet to decide on their choice of treatment, which may alter the distribution of choice of RRT.

## Conclusions

Based on our findings, a series of structured PDEP counseling sessions for patients progressing to ESRD can facilitate their selection of RRT. In a resource-limited setting, such as ours, where the number of patients who seek treatment for ESRD is rising, PDEP can provide adequate knowledge to persuade the patients towards the selection of a self-care RRT modality.
